# Re-creating reality: validation of fresh frozen full cadaver airway training with videolaryngoscopy and bougie FIRST strategy: The BOAH-course: a prospective, observational study

**DOI:** 10.1186/s13049-022-01006-4

**Published:** 2022-03-12

**Authors:** Sebastian Imach, Benny Kölbel, Andreas Böhmer, Dorothee Keipke, Tobias Ahnert

**Affiliations:** 1 Department of Trauma and Orthopedic Surgery, Cologne-Merheim Medical Center (CMMC), University Witten/Herdecke, Cologne, Germany; 2 Department of Surgery, Military Medical Center Ulm, Ulm, Germany; 3 Department of Anaesthesiology and Intensive Care Medicine, Cologne-Merheim Medical Center (CMMC), University Witten/Herdecke, Cologne, Germany

**Keywords:** Emergency airway management, Difficult airway, Intubation, Videolaryngoscopy, Bougie-assisted intubation, Fresh frozen cadaver training, HEMS, FPSR

## Abstract

**Background:**

Tracheal intubation is the gold standard in emergency airway management. One way of measuring intubation quality is first pass success rate (FPSR). Mastery of tracheal intubation and maintenance of the skill is challenging for non-anesthesiologists. A combination of individual measures can increase FPSR. Videolaryngoscopy is an important tool augmenting laryngeal visualization. Bougie-first strategy can further improve FPSR in difficult airways. Standardized positioning maneuvers and manipulation of the soft tissues can enhance laryngeal visualization. Fresh frozen cadavers (FFC) are superior models compared to commercially manufactured manikins. By purposefully manipulating FFCs, it is possible to mimic the pre-hospital intubation conditions of helicopter emergency medical service (HEMS).

**Methods:**

Twenty-four trauma surgeons (12 per Group, NOVICES: no pre-hospital experience, HEMS: HEMS physicians) completed an airway training course using FFCs. The FFCs were modified to match airway characteristics of 60 prospectively documented intubations by HEMS physicians prior to the study (BASELINE). In four scenarios the local HEMS airway standard (1: unaided direct laryngoscopy (DL), OLD) was compared to two scenarios with modifications of the intubation technique (2: augmented DL (bougie and patient positioning), 3: augmented videolaryngoscopy (aVL)) and a control scenario (4: VL and bougie, positioning by participant, CONTROL). FPSR, POGO score, Cormack and Lehane grade and duration of intubation were recorded. No participant had anesthesiological qualifications or experience in VL.

**Results:**

The comparison between CONTROL and BASELINE revealed a significant increase of FPSR and achieved C&L grade for HEMS group (FPSR 100%, absolute difference 23%, *p* ≤ .001). The use of videolaryngoscopy, bougie, and the application of positioning techniques required significantly more time in the CONTROL scenario (HEMS group: mean 34.0 s (IQR 28.3–47.5), absolute difference to BASELINE: 13.0 s, *p* = .045). The groups differed significantly in the median number of real-life intubations performed in any setting (NOVICES n = 5 (IQR 0–18.75), HEMS n = 68 (IQR 37.25–99.75)). In the control scenario no significant differences were found between both groups. The airway characteristics of the FFC showed no significant differences compared to BASELINE.

**Conclusion:**

Airway characteristics of a pre-hospital patient reference group cared for by HEMS were successfully reproduced in a fresh frozen cadaver model. In this setting, a combination of evidence based airway management techniques results in high FPSR and POGO rates of non-anesthesiological trained users. Comparable results (FPSR, POGO, duration of intubation) were achieved regardless of previous provider experience. The BOAH concept can therefore be used in the early stages of airway training and for skill maintenance.

## Background

Tracheal intubation is still the gold standard in often life-saving emergency airway management, despite the availability of alternatives [1, 2]. Since the probability of an unexpected difficult airway is increased in the pre-hospital setting, tracheal intubation must be mastered to a high standard by all clinicians, regardless of the main profession of the emergency personnel and the amount of anesthesiological training that may have been completed [3]. The quality of the airway management provided may be evaluated by the First Pass Success Rate (FPSR), defined as the proportion of properly placed tracheal tubes during the first laryngoscopic attempt. By prioritizing intubation success in the first attempt, complications such as swelling of the neck soft tissues, bleeding of the airway, hypotension, and, above all, hypoxia, may be avoided [4, 5].

The safe mastery of tracheal intubation and permanent maintenance of the skill is particularly challenging for non-anesthesiologists, since a minimum number of intubations (100–200 intubations) or a minimum duration of further training in anesthesiology or other emergency subspecialties is required to reach an acceptable level of expertise [6–9]. At the same time, it may be difficult for pre-hospital clinicians to get the exposure needed to maintain their skills in clinical procedures such as tracheal intubations, solely from their pre-hospital work [10, 11].

Various individual measures or a combination of individual measures can increase FPSR in emergency airway management [12, 13]. Videolaryngoscopy appears to augment laryngeal visualization [14–17]. Furthermore the use of a bougie-first strategy can achieve up to 14% improved FPSR in patients with difficult airways [18, 19] Standardized positioning maneuvers considering cervical spine protection, such as raising the upper body by 25° (ramping) and elevating the head can further increase laryngeal visibility [20–24]. Selective manipulation of the soft tissues of the neck during bimanual laryngoscopy can improve laryngeal visualization up to 25% [25, 26].

For advanced airway management training simulation appears to be more effective than non-simulation education (including OR training) [27, 28]. Biological models like fresh frozen cadavers are perceived as more realistic in terms of intubation conditions, learner satisfaction is increased in a cadaver model compared to manikins (Standardized Mean Difference (SMD) 0.79; 95% CI 0.49, 1.08) and outcomes in real clinical settings tend to favor a cadaver model (SMD 0.28; 95%: −0.62, 1.18) [27]. At the same time, it increases user confidence and reduces the duration of airway management [29–31]. Acquisition of non-time dependent skills may be facilitated by advanced airway manikins (SMD −1.10; 95%, − 2.06, − 0.13) [32]. A combination of both advanced manikins and cadavers is possible [33, 34]. Paramount for learning success is task repetition with intermittent feedback [35, 36].

The combination of 4 h manikin training, a video study, and 2 h cadaver training leads to an equalization of POGO scores between inexperienced and experienced users. These in vitro results showed a correlation of FPSR in real critical care patients [27]. The POGO score indicates the estimated percentage of glottis opening visualized during laryngoscopy by the clinician [37, 38]. By purposefully manipulating the cadaver, it is possible to mimic prehospital intubation conditions and train operators accordingly. By using Peyton´s four-step-approach for teaching manual skills including skill deconstruction to the smallest teachable unit (micro-teaching), providers are able to go through different stages of skill acquisition in a compressed way, which finally enables training in the form of deliberate practice [39–42].

Equipping local HEMS with VL for the first time while employing clinicians with limited airway management experience made the development of an effective training concept necessary integrating VL.

Our first hypothesis is that the proposed combination of individual techniques to improve intubation conditions will allow a high FPSR for the non-anesthesiology trained user (less than 100 intubations in the operating room setting) in a realistic cadaver model. Secondly, we hypothesize that FPSR, the duration of intubation, and the POGO scores between experienced users (trauma surgery fellows and HEMS physicians) and NOVICES (trauma surgery residents) would converge over the course of training and would, over time, no longer differ significantly. Third, the present fresh frozen cadaver model would show a high correlation with real pre-hospital intubation conditions.

## Methods

### Study population

The study population was composed of 2 groups with 12 physicians each. All 24 participants were in different stages of the same trauma surgery residency program (Department of Traumatology and Orthopedic Surgery Cologne-Merheim Medical Center (CMMC), Cologne, Germany), or had already successfully completed it. No participants had any previous anesthesiological qualifications. In Germany, a trauma surgery residency lasts 6 years and is undertaken after completion of medical school (6 years). The program consists of an intensive care rotation (ICU) with a length of at least 6 months.

The first group (NOVICES) was in the first 2 years of the program. ICU rotation was not mandatory, and they had not received separate airway training at any time before the study. This group was not regarded to be competent in airway management but was chosen to determine the effect of the course concept on the very early stages of intubation skill acquisition.

The second group (HEMS) was formed by the physicians of the local HEMS service. The helicopter has been equipped with a videolaryngoscope after the study.

In order to be deployed as a pre-hospital emergency physician in local HEMS, one must have a board-certified qualification, which requires 6 months of work in an ICU, 80 h of theoretical teaching, and completion of 50 pre-hospital emergency missions accompanied by an experienced pre-hospital emergency physician. In addition, according to the local protocol, a 6-month full-time rotation as a pre-hospital emergency physician (at least 300 missions), a 2-week rotation in pediatric anesthesia, a standardized course on trauma care (ATLS®), and an intensive care transport course must be completed. Thereafter, an annual continuing education requirement must be met.

### Design

Four weeks before the start of the study, all participants received standardized digital preparatory materials consisting of theoretical journal articles (regarding FPSR), instructional videos (landmarks intubation), and manufacturer's instructions (bougie and videolaryngoscope), which had to be completed by the start of the study (duration approximately 3 h). All contents were summarized by the study director (IS) in a 45 min lecture before the start of the study. Afterwards, all airway techniques were trained individually and in complete sequence by the study instructors (AT, BK, IS) on a Manikin (Deluxe Difficult Airway Trainer, Laerdal, Starvanger, Norway), and all questions raised by the participants were answered. Each participant was supported in a standardized manner by a flight-paramedic during the training. These paramedics regularly serve as technical crew members in local HEMS (HEMS-TC).

One day before the training, participants were given a pre-questionnaire (12 questions, intubation experience), and 1 day after the training, a post-questionnaire (9 questions, improvement of skills and perception of FFC). Five-point Likert scales were used in the questionnaires (1 = no agreement with the statement, 5 = maximum agreement with the statement).

### Setting

The participants were distributed by lot to 2 training dates. On each date, 5 fresh frozen whole body human cadavers (FFC) were available. All cadavers were thawed in a standardized manner 24 h before the start of training. The 5 FFC mimicked a typical prehospital patient cohort of local HEMS in terms of characteristics relevant for airway management. For this purpose, difficult airway characteristics (e.g., mouth opening, Mallampati score, neck mobility, presence of airway obstruction, thyromental distance, LEMON score [43, 44]) were documented prospectively from 07/2019 to 07/2020 in 60 pre-hospital intubations (BASELINE). For all FFC, the best possible Cormack and Lehane score was obtained by 2 board-certified anaesthetists (female KD and male BA) through direct laryngoscopy without time limitation before the start of training [33]. FFC were not modified after that, beside limiting mouth opening of the 4th FFC to 3 cm and limiting neck mobility of the 5th FFC to 15° extension to reflect typical criteria characterizing a potentially anatomically difficult airway in BASELINE.

### Airway training/scenarios

**Fig. 1:**
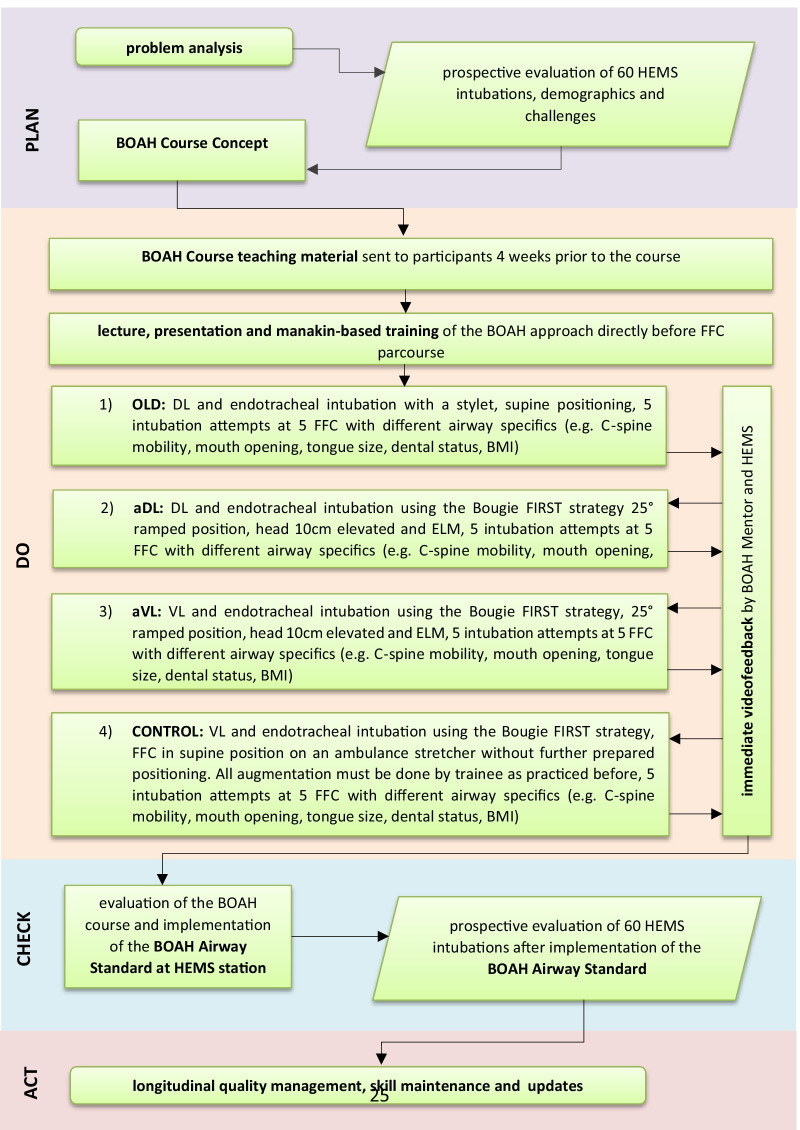
Flowchart of the BOAH-Airway-Project, outlining the planning, execution and evaluation of the BOAH-Airway-Concept and implementation of the BOAH-Airway-Standard at Christoph 3 HEMS. (HEMS, helicopter emergency medical service; BOAH, best of airwaymanagement in HEMS; ELM, external laryngeal manipulation; FFC, fresh frozen cadavers; OLD, airway standard Christoph 3 prior to BOAH project; aDL, augmented direct laryngoscopy; aVL, augmented videolaryngoscopy; BMI, body-mass-index)

All participants had to undergo the 4 scenarios described below (Fig. 1). In each scenario, participants had one intubation attempt per FFC (5 intubations/scenario).

An intubation attempt was defined as any insertion of a laryngoscope beyond the teeth, whether an endotracheal tube (ETT) was passed or not. First-attempt success was defined as a properly placed tracheal tube with an inflated cuff during the first laryngoscopic attempt as confirmed by videolaryngoscopy (VL). There was no time limit for an attempt. Instructors rated the intubation technique on a 10-point scale for each trial, with 10 being the highest.

Scenario 1 "unaided direct laryngoscopy (OLD, uDL)": DL with a Macintosh-like blade (Size 4) with an endotracheal tube (Size 7.5) and stylet. FFC in supine position on an ambulance stretcher without further prepared positioning. This setting represents the local HEMS airway standard prior to the start of training. The scenario was used to validate the model by comparison with real life BASELINE data.

Scenario 2 "augmented direct laryngoscopy (aDL)": DL with a MacIntosh-like blade (Size 4) using the Bougie FIRST strategy (S-Guide® 15Fr 65 cm, VBM Medical, Sulz a.N., Germany). FFC with the upper body in a 25° ramped position, the head was elevated by 10 cm, and external laryngeal manipulation (ELM) was advised.

Scenario 3 "augmented videolaryngoscopy (aVL)": VL (GlideScope® Go™, Verathon, Bothell, USA) with a MacIntosh-like blade (Size 4) using the Bougie FIRST strategy. FFC with the upper body in a 25° ramped position, the head was elevated by 10 cm, and external laryngeal manipulation (ELM) was advised.

Scenario 4 "augmented videolaryngoscopy with self-positioning (CONTROL)": VL with a MacIntosh-like blade (Size 4) using the Bougie FIRST strategy. FFC lying in supine position on an ambulance stretcher without further prepared positioning. All augmentation maneuvers have been allowed. This setting represents our new evidence based and best trained local HEMS airway standard after the introduction of the VL (Best of airway management in HEMS, BOAH).

### Data collection and analysis

All study data (FPSR, duration of intubation, C&L grade, POGO-Score) were collected in real time by AT, KB, and IS, and immediately documented on a standard form. After each scenario, the data were transferred to a spreadsheet program (Microsoft Excel, version 365, Redmond, USA).

Statistical analysis was performed using SPSS statistical software (version 27; IBM Inc., Armonk, NY, USA). Data are presented as absolute and relative values, or as means with standard deviations (SD), respectively. In case of skew distributed data, medians with inter-quartile ranges (IQR) were used instead of means. Group comparisons were performed with a Chi-squared test in the case of categorical data, and the Mann–Whitney U test for continuous variables. The Kruskall-Wallis test was used for simultaneous significance testing of several groups.

Correlation calculation was done for non-metric scaled variables according to Spearman (Rho).

The level of significance was defined at *p* < 0.05.

### Study approval

The present study was approved by the ethics committee of the Faculty of Medicine, University of Witten/Herdecke, Germany (No. 86/2019), with a Study Register No. German Trail Register (DRKS00024125).

## Results

Baseline data of the NOVICES and HEMS group regarding professional experience and intubation skills significantly differ as expected (Table 1).

**Table 1:** Characteristics of participants

	Novice n = 12	HEMS n = 12	
Professional years as doctor, median IQR	2 (1–4)	7 (6–7.75)	< .001
Formal preclinical qualification (n, %)	3 (25.0%)	12 (100%)	
Professional years as emergency physician median, IQR	0 (0–0.75)	4 (3.25–5)	< .001
Formal clinical intubation training (n, %)	2 (16.7%)	3 (25.0%)	
Real-life intubations total, median, IQR	5 (0–18.75)	68 (37.25–99.75)	< .001
Intubations in hospital, median, IQR	5 (0–11)	7 (0–42.5)	.551
Intubations per year preclinical, median, IQR	0 (0–0)	10 (7.25–15)	< .001
Intubations by videolaryngoskop total, median, IQR	0 (0–1)	3 (1.25–10.5)	.002
Intubations using bougie total, median, IQR	0 (0–1)	4 (1–9)	< .001
Cricothyroidotomy performed total, median, IQR	0 (0–0)	0 (0–0.75)	.551

IQR, Interquartile range

In the CONTROL scenario, testing the main hypothesis, the FPSR and achieved C&L grade of the HEMS group increased significantly compared to the pre-hospital patient reference group BASELINE (HEMS FPSR 100%, absolute difference: 23%, *p* ≤ 0.001; median C&L 1, IQR 1–2, *p* = 0.018). Compared to BASELINE the use of videolaryngoscopy, bougie, and the application of positioning techniques required significantly more time (median HEMS 34.0 s, IQR 28.3–47.5, absolute difference: 13.0 s, *p* = 0.045) (Table 2).

**Table 2:** Group comparison intubation performance

	NOVICES group n = 60	HEMS group n = 60	*p*-value
**Unaided direct laryngoscopy (OLD, uDL)**			
uDL FPSR (n, %)	48 (80.0%)	50 (83.3%)	.638
uDl C&L grade (n, median, IQR)	2 (1–2)	2 (1–2)	.158
uDL POGO-Score (%, median, IQR)	61.3 (30.0–100.0)	80.0 (50.0–100.0)	.104
uDL time intubation (s, median, IQR)	22.0 (16.0–34.6)	21.0 (12.3–32.6)	.339
**Augmented direct laryngoscopy (aDL)**			
aDL FPSR (n, %)	56 (93.3%)	57 (95.0%)	.698
aDL C&L grade (n, median, IQR)	2 (1–2)	1 (1–2)	.119
aDL POGO-score (%, median, IQR)	80.0 (80.0–100.0)	100.0 (100.0–100.0)	.003
aDL time intubation (s, median, IQR)	32.0 (25.0–41.5)	26.0 (19.3–34.8)	< .001
**Augmented videolaryngoscopy (aVL)**			
aVL FPSR (n, %)	59 (98.3%)	60 (100.0%)	.317
aVL C&L grade (n, median, IQR)	1 (1–2)	1 (1–2)	.842
aVL POGO-Score (%, median, IQR)	100.0 (90.0–100.0)	100.0 (90.0–100.0)	.626
aVL time intubation (s, median, IQR)	32.0 (24.25–40.0)	24.0 (19.3–30.0)	< .001
**Augmented videolaryngoscopy with self-positioning (CONTROL)**			
CONTROL FPSR (n, %)	60 (100.0%)	60 (100.0%)	1.000
CONTROL C&L grade (%, median, IQR)	1 (1–2)	1 (1–2)	.593
CONTROL POGO-Score (%, median, IQR)	100.0 (100.0–100.0)	100.0 (80.0–100.0)	.427
CONTROL time intubation (s, median, IQR)	30.5 (27.0–44.8)	34.0 (28.3–47.5)	.370

FPS, first pass success; C&L, Cormack & Lehane grade; POGO score, percentage of glottis opening

For the secondary hypothesis of converging intubation performance (FPSR, POGO, duration of intubation) a group comparison between NOVICES and HEMS of the CONTROL scenario, in which all maneuvers were performed independently by the participants, found no significant differences. In the OLD scenario as standard of care the HEMS group still scored higher in intubation conditions (C&L grade, POGO) without being statistically significant. While FPSR or intubation duration already demonstrated no significant difference. The quality of intubation performance in OLD was rated significantly lower by instructors in the NOVICE group (NOVICE 7.0 (IQR 5.25–8.00), HEMS 8.0 (7.0–10.0), *p* < 0.001), while in CONTROL no difference was found. The data of the four study scenarios are shown in Table 2.

In order to verify the third hypothesis, a comparison of the intubation characteristics of the two FFC groups and the pre-hospital patient reference group (BASELINE) showed no statistically significant differences between the 3 groups (Table 3). Accordingly, There was no significant difference of intubation performance by the HEMS group between the OLD scenario and the BASELINE data (FPSR n = 47 (77%, *p* = 0.317, C&L grade median 2 (IQR 1–2, *p* = 0.124), duration of intubation median 25.5 s (IQR 18.5–45.0, *p* = 0.994). The perceived realism of the FFC was rated higher by the HEMS group (NOVICES median 4.0 (IQR 3.6–4.0), HEMS median 5.0 (IQR 5.0–5.0), *p* < 0.001).

**Table 3:** Cadaver specifications

	HEMS group n = 60	FFC group 1 n = 5	FFC group 2 n = 5	*p*-value
Sex (female, n, %)	44 (73.3%)	2 (40.0%)	1 (20.0%)	
Age (years, median, IQR)	65.0 (50.5–80.75)	78 (72.5–85.50)	78 (68.5–80.5)	.130
Body weight (kg, median, IQR)	80.0 (75.0–90.0)	68 (47.6–85.1)	72.6 (61.5–90.9)	.186
Body height (cm, median, IQR)	178.5 (170.0–185.0))	175.3 (162.6–177.8)	177.8 (165.1–179.1)	.387
Body Mass Index (kg/m^2^, median, IQR)	26.1 (23.4–27.8)	21.5 (17.5–28.6)	23.2 (20.8–31.0)	.269
Dental status intact (n, %)	42 (70.0%)	2 (40.0%)	2 (40.0%)	.087
Mouth opening (cm, median, IQR)	4.0 (3.0–4.0)	3.5 (2.75–3.75)	3.5 (2.75–4.0)	.556
Thyreomentale distance (cm, median, IQR)	7.0 (6.0–7.5)	7.0 (6.5–7.75)	7.0 (6.75–7.75)	.364
Max. neck extension (> 30°, n, %))	37 (61.7%)	4 (80.0%)	4 (80.0%)	.510
C&L grade in field (direct laryngoscopy, median, IQR)	2 (1–2)			
Best C&L grade by anaesthetist in lab (direct laryngoscopy, median, IQR)		1 (1–2)	1 (1–2)	
POGO score by anaesthetist in lab (direct laryngoscopy, median, IQR)		100% (77.5–100)	100% (80–100)	

C&L, Cormack & Lehane grade; POGO score, percentage of glottis opening

For the NOVICES group, there was no significant correlation between the number of intubations performed on a real patient prior to the start of training and FPSR in the OLD and CONTROL scenarios (OLD Rho 0.178, *p* = 0.172; CONTROL Rho − 0.212, *p* = 0.105); this correlation is also absent in the HEMS group (OLD Rho − 0.104, *p* = 0.430; CONTROL Rho − 0.094, *p* = 0.473).

## Discussion

In this study, a combination of positioning maneuvers and the use of technical devices i.e. videolaryngoscopy and bougie FIRST strategy succeeded in significantly increasing the FPSR by at best 23% in a fresh frozen cadaver model.

As shown by Driver et al. in a single center randomized controlled trial at an emergency department in Minneapolis, the bougie FIRST strategy alone is a suitable tool to significantly increase the FPSR in adult emergency patients [18]. Among 380 patients with at least 1 difficult airway characteristic (body fluids obscuring the laryngeal view, airway obstruction or edema, obesity, short neck, small mandible, large tongue, facial trauma, or the need for cervical spine immobilization) first-attempt intubation success was higher in the bougie group (96%) than in the endotracheal tube + stylet group (82%) (absolute difference, 14% [32]). VL used by US emergency physicians in the 3rd year of training raises FPSR up to 90% compared to DL (FPSR 73%). Therefore VL is recommended as the primary intubating device for patients with difficult airway characteristics [45]. A RCT in in-hospital inducation of anaesthesia showed a higher FPSR in VL compared to DL ((93% vs. 84%), *p* = 0.026), with at the same time better visualization of the vocal cord level (C&L I/II: 93% vs. 81%, *p* < 0.01) [46]. POGO scores also improved in our study by using VL. Those high FPSR rates could be reached in different HEMS settings with both an anesthesiological or trauma surgical specialization by using videolaryngoscopy as first line device [16, 47].

A better visualization of the vocal cord level results in higher FPSR rates in patients with cervical immobilization representing a difficult airway characteristic what is relevant for a trauma patient population [48].

Improving visualization and adjustability of the vocal cords by using VL may be at the expense of a longer intubation time compared to DL (46 vs. 33 s, absolute difference 13 s, *p* < 0.001) [46]. The combination of VL and bougie FIRST strategy used in the study also required significantly more time (absolute difference 13.0 s) compared to DL, but participants were not trained in VL use before. The clinical significance of this difference must be questioned when considering the time to desaturation demonstrated in previous studies. For example, saturations < 90% rarely occur after 3 min of preoxygenation with an intubation time of less than 60 s [49].

Comparable results (FPSR, POGO, intubation time) were achieved by both groups regardless of previous intubation experience. Numerous studies on real life patients have shown the correlation of the FPSR with the number of intubations already performed by the provider. The learning curve starts flattening only after 25–30 intubations [6, 50]. The BOAH course concept therefore seems suitable to move the first part of this learning curve from a real life scenario into the safe and reproducible laboratory environment providing a starting point for the necessary real-life experience. Especially for non- anesthesiological trained users, this training environment offers the conditions for rapid task repetition and gain of experience with devices like the videolaryngoscope and the bougie.

The cadaver model successfully reproduced the airway characteristics of a pre-hospital patient group cared for by HEMS in the lab setting. Thereby, FPRS, duration of intubation, and C&L grade showed no significant differences between the lab setting and the pre-hospital setting.

### Limitations

FFC can only represent the anatomical aspects of airway management. In real life, the safe application of emergency anesthesia with the use of muscle relaxants is a key element in creating optimal intubation conditions. Mastery of this skill did not need to be demonstrated by the participants.

The widespread use of FFC is limited by costs, logistical requirements such as cooling and complex supply (relevant delivery time) although they are commercially available. They can only be refrozen once.

The study lacks an anesthesiological trained comparison group. However, considering the high rate of FPSR, we presume no significant difference in the FPSR rate. However, anesthesia professionals might perform intubation in less time and with optimal ergonomics.

A prospective evaluation of the FPSR in the HEMS setting after standardized airway course using the BOAH concept should be performed to validate the findings in real life.

## Conclusion

In a fresh frozen cadaver model, a combination of evidenced based airway management techniques results in high FPSR and POGO rates of non-anesthesiological trained users. Using teaching concepts like deliberate practice, immediate video-feedback-practice loops, 1:1 teaching with a mentor enables single technique mastery regardless of previous provider experience. The cadaver model successfully reproduced airway characteristics of a pre-hospital patient reference group cared for by HEMS. The BOAH concept can move the early learning curve of emergency intubation training from a real life scenario to a controlled high-volume laboratory setting and can therefore be used in the early stages of airway training.

## Data Availability

Please contact author for data requests.

## Abbreviations

aDL: Augmented direct laryngoscopy
ATLS: Advanced trauma life support
aVL: Augmented videolaryngoscopy
BMI: Body mass index
BOAH: Best of airway management in HEMS
C&L: Cormack & Lehane grade
ELM: External laryngeal manipulation
FFC: Fresh frozen cadavers
FPSR: First pass success rate
HEMS: Helicopter emergency medical service
IQR: Interquartile range
OLD: Airway standard Christoph 3 prior to BOAH project
POGO score: Percentage of glottis opening
